# Supplementary material for: Degradation of Styrenic Plastics during Recycling: Accommodation of PP within ABS after WEEE Plastics Imperfect Sorting
Source: Polymers (Basel). 2021 Apr 29;13(9):1439. doi: 10.3390/polym13091439 (PMC8124459; doi:10.3390/polym13091439)
Supplement: Supplementary file 1 [file polymers-13-01439-s001.zip › polymers-1180993-supplementary.pdf]

Degradation of styrenic plastics during recycling: Accommodation of PP within ABS after WEEE plastics imperfect sorting

Supporting information

Content

List of figures 1

A. Appendix to “2. Materials and methods” 3

B. Appendices to “3.1 Impact of PP on ABS at varying incorporation rates” 4

3.1.1 Impact properties 4

3.1.3 Morphology 6

C. Appendices to “3.2 Compatibilizers preselection” 7

3.2.1 Impact properties 7

3.2.2 Morphology 10

3.2.3 Tensile properties 11

D. Appendices to “3.3 D.O.E.-R.S.M.: Influence of process parameters and interactions with compatibilizers toward impact properties” 12

List of figures

Figure S1: Comparison of force-displacement curves of instrumented notched Charpy impact at 0.8 m/s (left) and 2.9 m/s (right) – uncontaminated and contaminated with 4w% PP ABS – 5 specimens / batch 3

Figure S2: Force-displacement curves of unnotched Charpy impact on virgin ABS, SAN (both directly injected from pellets) and ABS contaminated with 6w% PP 4

Figure S3: G-Moduli extracted from unnotched Charpy test versus E-Moduli from tensile tests of ABS progressively contaminated with PP – dots represent means on 6 to 10 specimens, errors bars represent standard deviations 5

Figure S4: SEM pictures ABS + 4 w% PP dogbones cryofractured at 90° (top) & 45° (bottom) – 5000 x and 25000 x magnifications 6

Figure S5: FT-IR ATR spectra of ABS (black), PP (blue) and ABS + 8w% PP (red)................................... 6

Figure S6: Force-displacement curves of notched Charpy impact – virgin extruded ABS batch “Vext”, 4w% PP contaminated batch “P4” and separately additived batches with 1, 2 & 3 phr of PPH-g-MA, PPC-g-MA, ABS-g-MA, TPE-g-MA, SEBS and PP-g-SAN 7

Figure S7: Force-displacement curves of unnotched Charpy impact – same batches and denomination as previous figure 8
Figure S8: Force-displacement curves of unnotched Charpy impact of ABS contaminated with 4w% PP and additived with 1, 2 & 3 phr SEBS (a plot for each rate) and picture of 2 phr SEBS specimens after impact – green curves and frame for partially broken specimens... 9
Figure S9: SEM pictures of cryofractured dogbones – effect of compatibilizers at 3 phr on a 4w% PP contamination – 25000 x magnification... 10
Figure S10: Tensile tests of compatibilization trials with PP-g-MA and SEBS of ABS contaminated with 4w% of PP – magnification on stress peak -"Vext" for virgin and extruded ABS, other batches with 4w% PP – phr rates correspond to PP-g-MA and SEBS 11
Figure S11: SEM pictures of cryofractured dogbones of ABS contaminated with 4 w% PP – different extrusion temperatures and screw speeds.. 12
Figure S12: SEM pictures of cryofractured dogbones of ABS + 4 w% PP – 0.5 phr PP-g-SAN, 200°C and 200 rpm – near the surface, elongated phases framed in pink 13
Figure S13: SEM pictures of cryofractured dogbones of ABS + 4 w% PP – 0.5 phr PP-g-SAN, 240°C and 300 rpm – near the surface, elongated phases framed in pink 14
Figure S14: Notched Charpy impact break energies – D.O.E.-R.S.M. experimental values – ABS + 4w% PP without compatibilizer or with SEBS, PP-g-SAN or PP-g-MA – abscissa axis indicates extrusion temperature (°C), screw speed (rpm) and compatibilizer loading rate (phr) 15
Figure S15: Unnotched Charpy impact break energies – D.O.E.-R.S.M. experimental values – samples and designation identical to previous figure.. 15
Figure S16: Response Surface Methodology applied to notched Charpy break energy of ABS + 4w% PP system - uncompatibilized, additived with SEBS, PP-g-MA or PP-g-SAN – additive concentration (1-3 or 0.5-1.5 phr), extruder temperature (200-240°C) and screw speed (200-300 rpm) as variables – lower color scale for PP-g-SAN (values in orange) 16
Figure S17: Response Surface Methodology applied to unnotched Charpy break energy of ABS + 4w% PP system - uncompatibilized, additived with SEBS, PP-g-MA or PP-g-SAN – additive concentration (1-3 or 0.5-1.5 phr), extruder temperature (200-240°C) and screw speed (200-300 rpm) as variables – lower color scale for PP-g-SAN (values in orange) 17

A. Appendix to "2. Materials and methods"

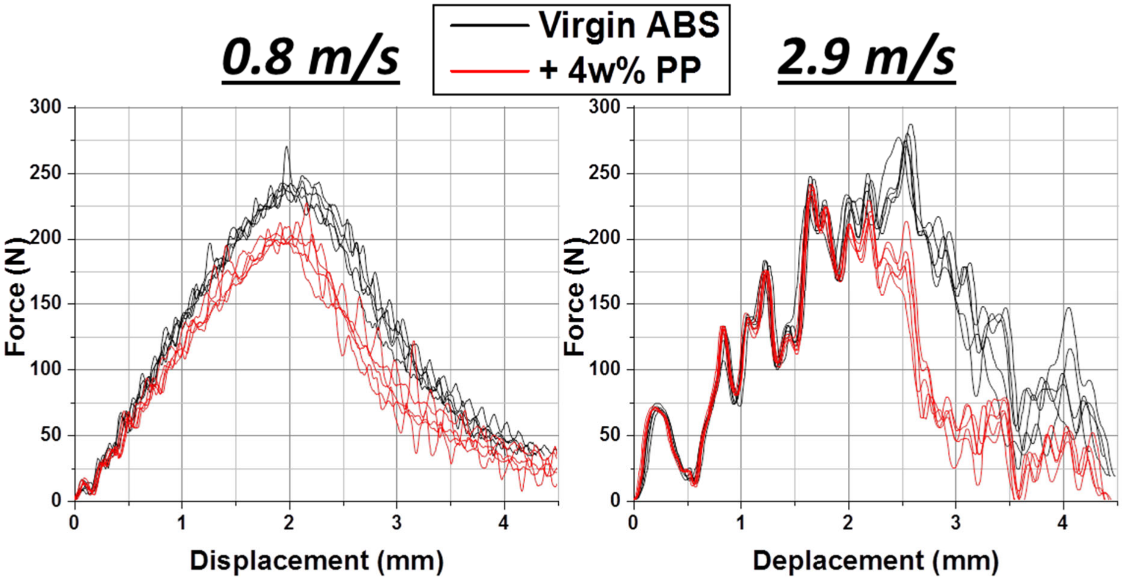

Figure S1: Comparison of force-displacement curves of instrumented notched Charpy impact at 0.8 m/s (left) and 2.9 m/s (right) – uncontaminated and contaminated with 4w% PP ABS – 5 specimens / batch

B. Appendices to "3.1 Impact of PP on ABS at varying incorporation rates"

3.1.1 Impact properties

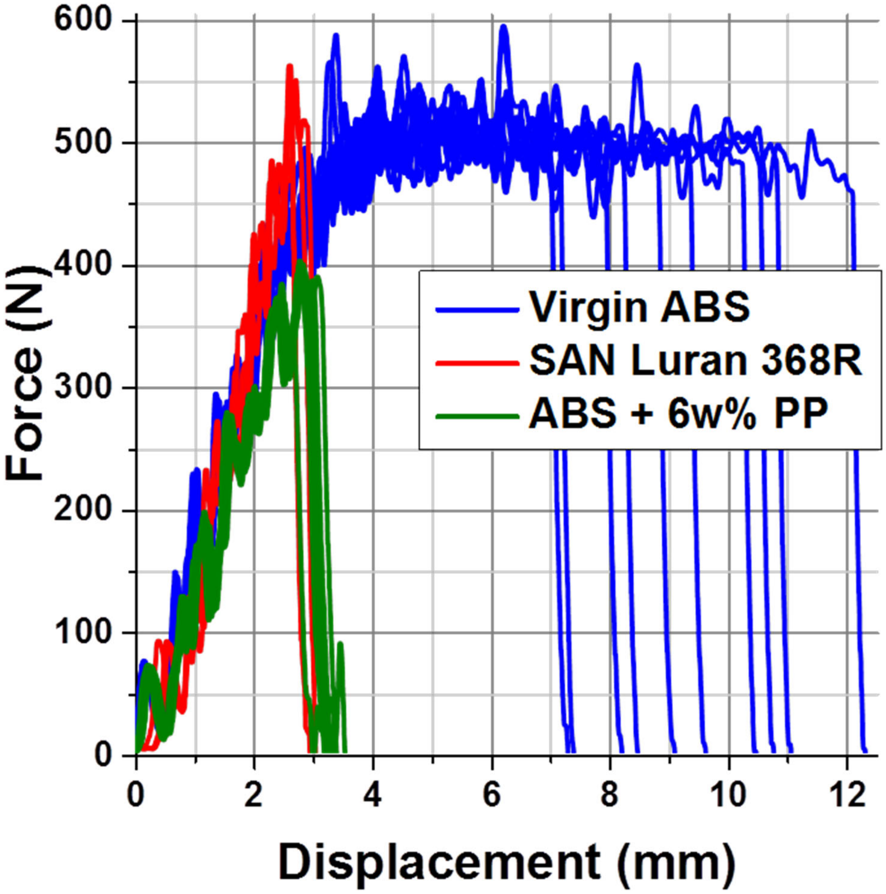

Figure S2: Force-displacement curves of unnotched Charpy impact on virgin ABS, SAN (both directly injected from pellets) and ABS contaminated with 6w% PP

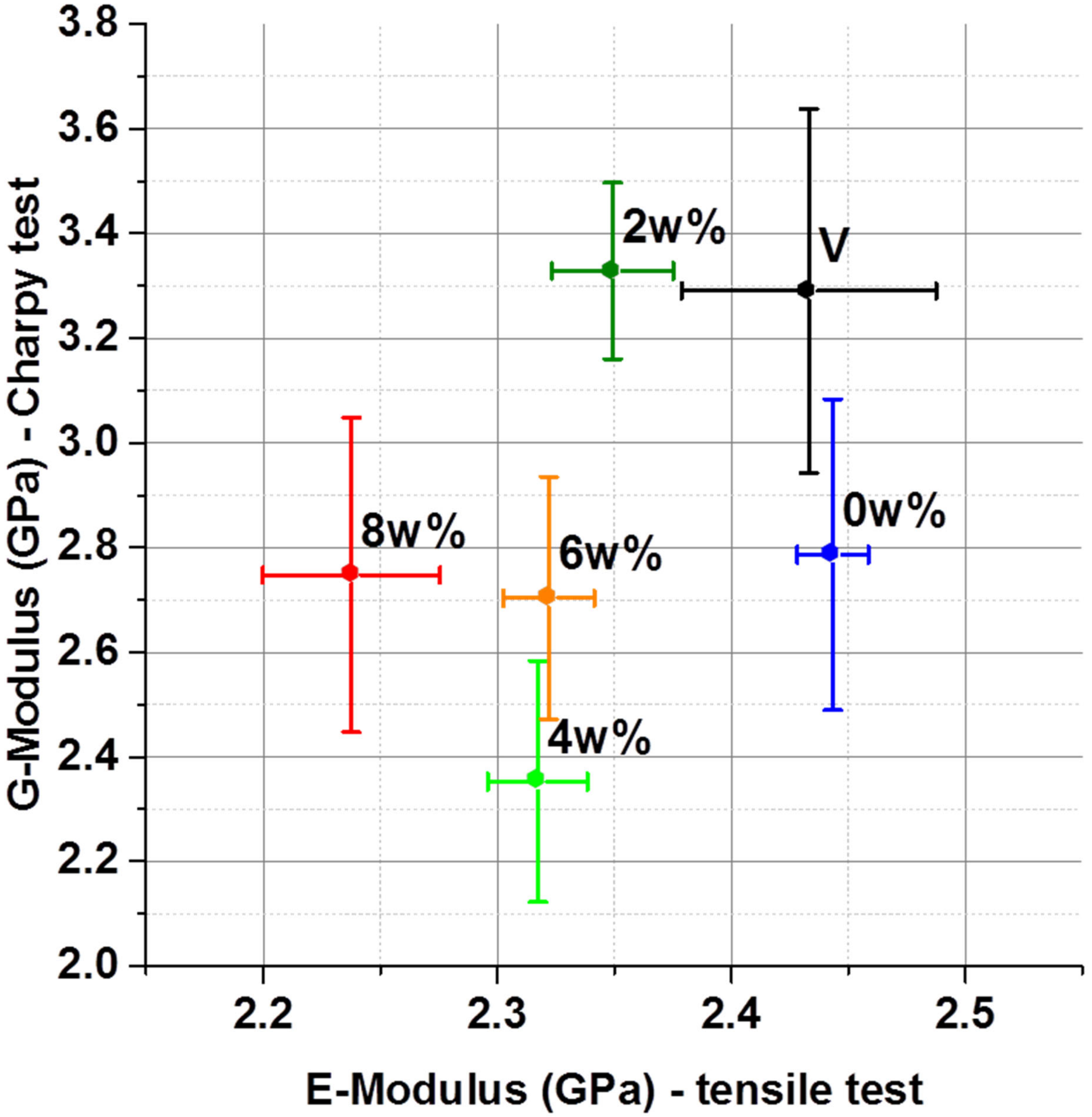

Figure S3: G-Moduli extracted from unnotched Charpy test versus E-Moduli from tensile tests of ABS progressively contaminated with PP – dots represent means on 6 to 10 specimens, errors bars represent standard deviations

3.1.3 Morphology

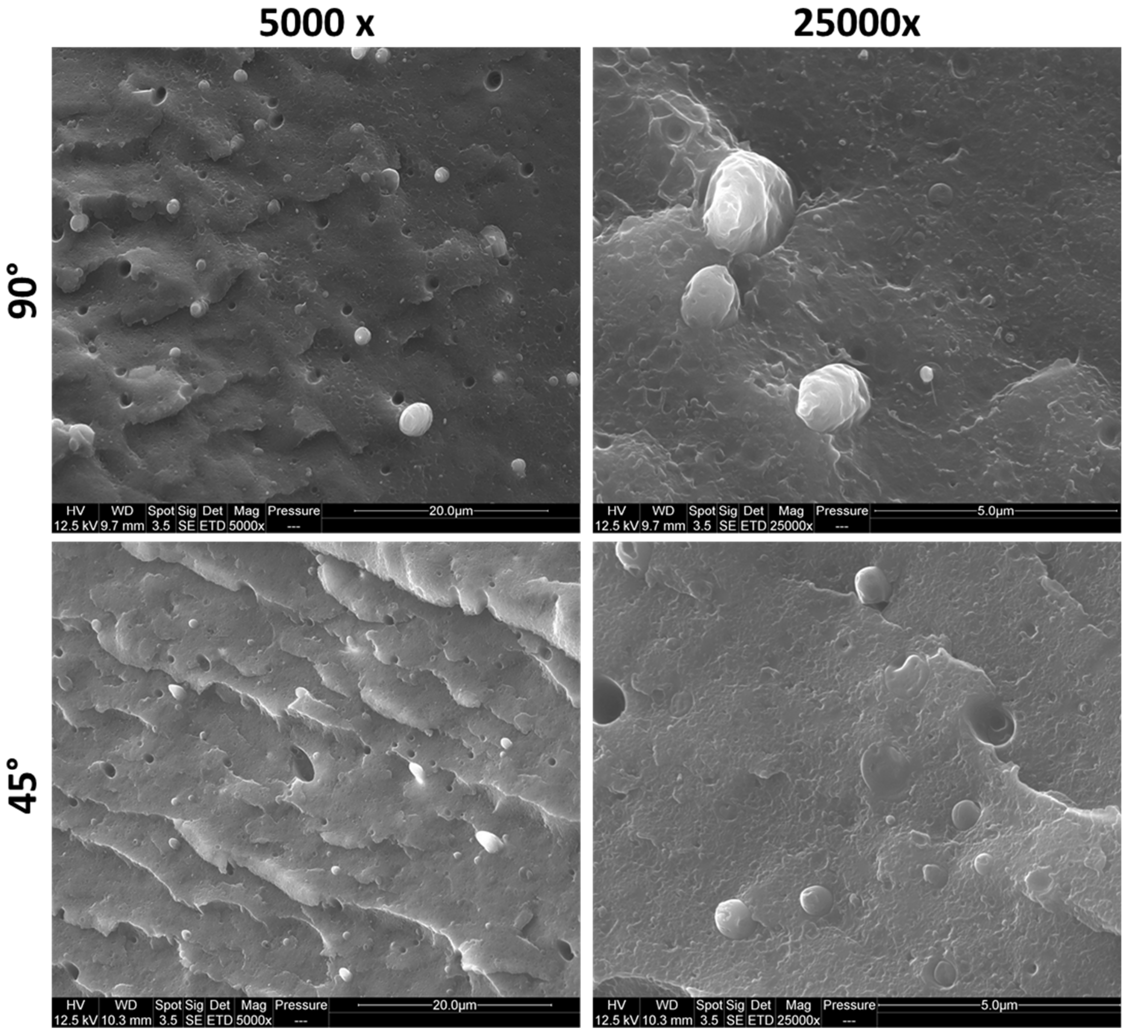

Figure S4: SEM pictures ABS + 4 w% PP dogbones cryofractured at 90° (top) & 45° (bottom) – 5000 x and 25000 x magnifications

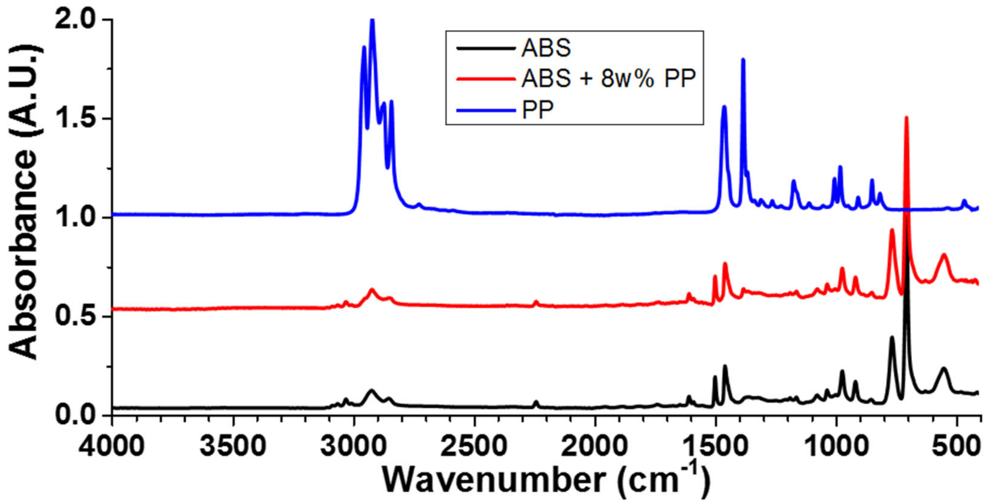

Figure S5: FT-IR ATR spectra of ABS (black), PP (blue) and ABS + 8w% PP (red)

C. Appendices to “3.2 Compatibilizers preselection”

3.2.1 Impact properties

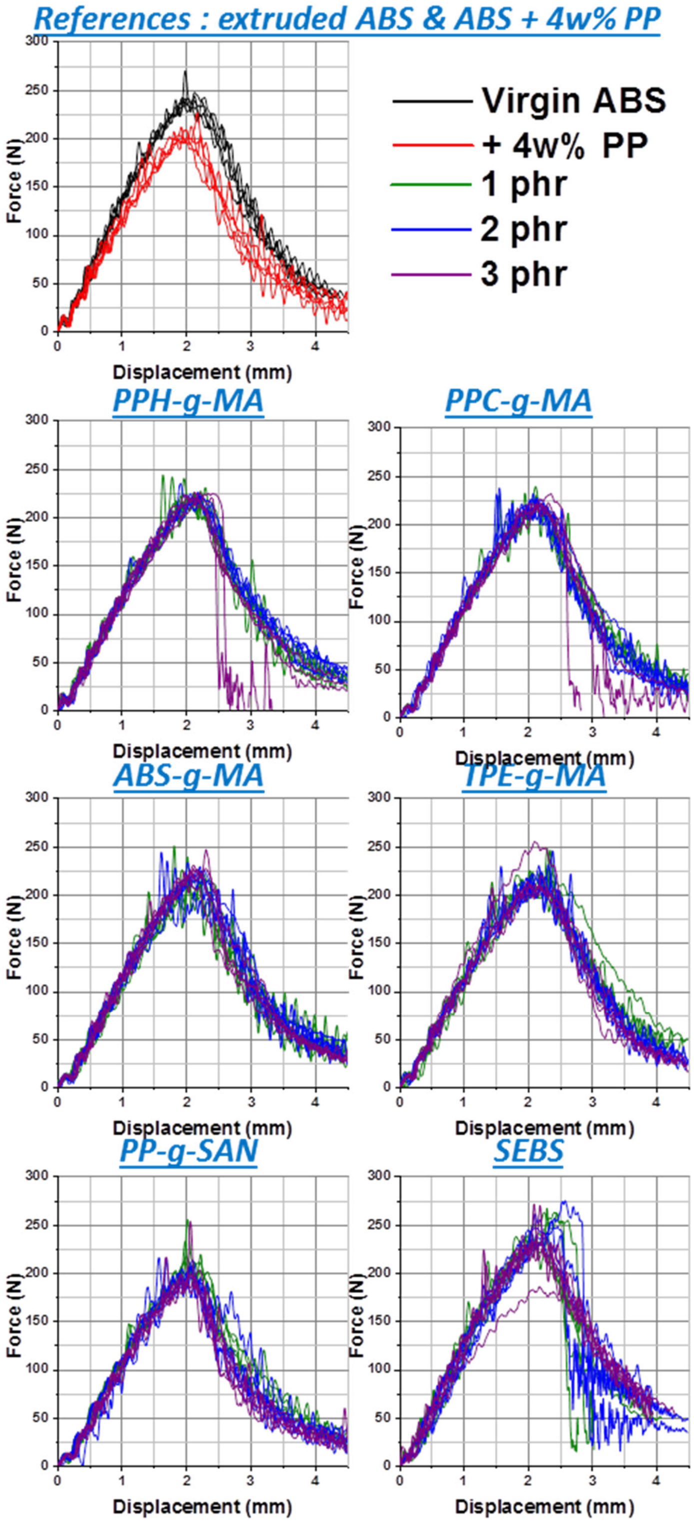

Figure S6: Force-displacement curves of notched Charpy impact – virgin extruded ABS batch “Vext”, 4w% PP contaminated batch “P4” and separately additived batches with 1, 2 & 3 phr of PPH-g-MA, PPC-g-MA, ABS-g-MA, TPE-g-MA, SEBS and PP-g-SAN

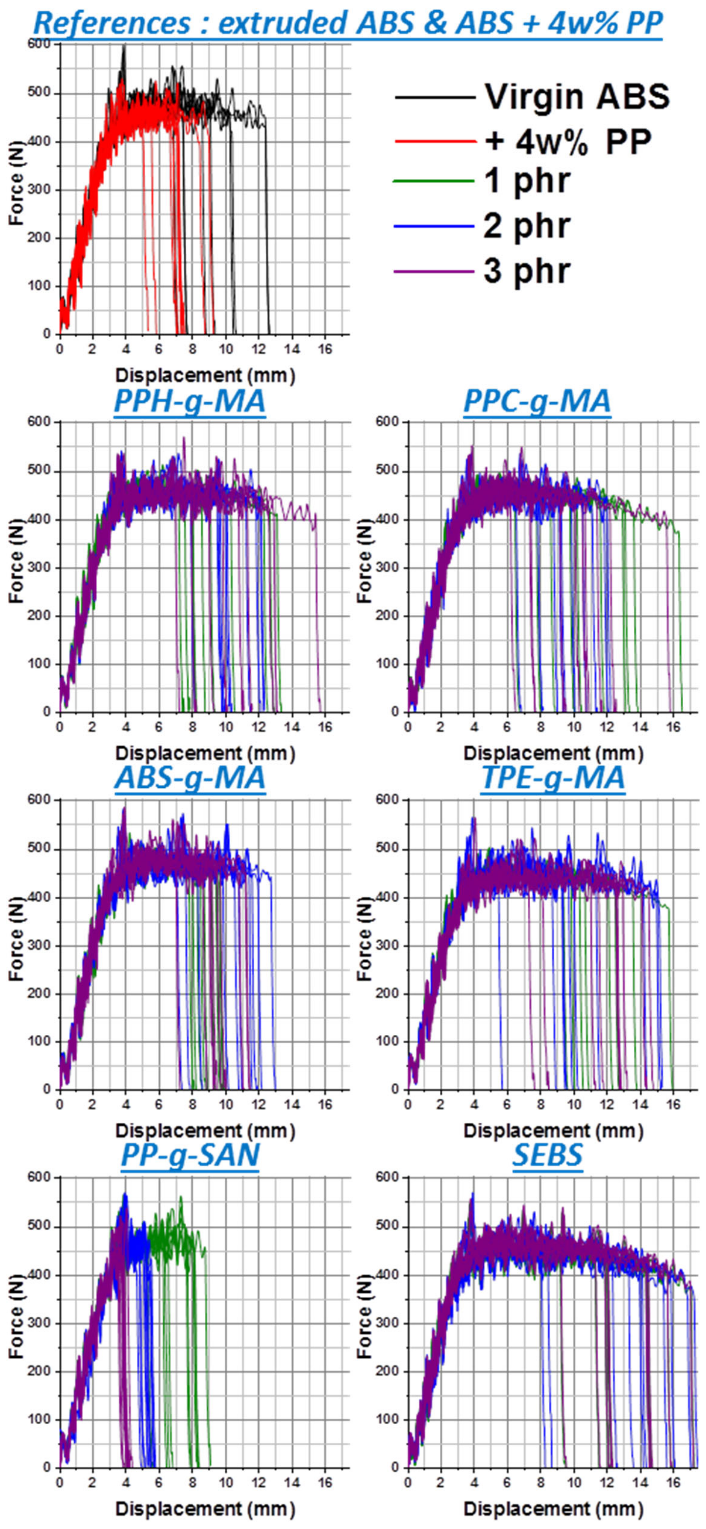

Figure S7: Force-displacement curves of unnotched Charpy impact – same batches and denomination as previous figure

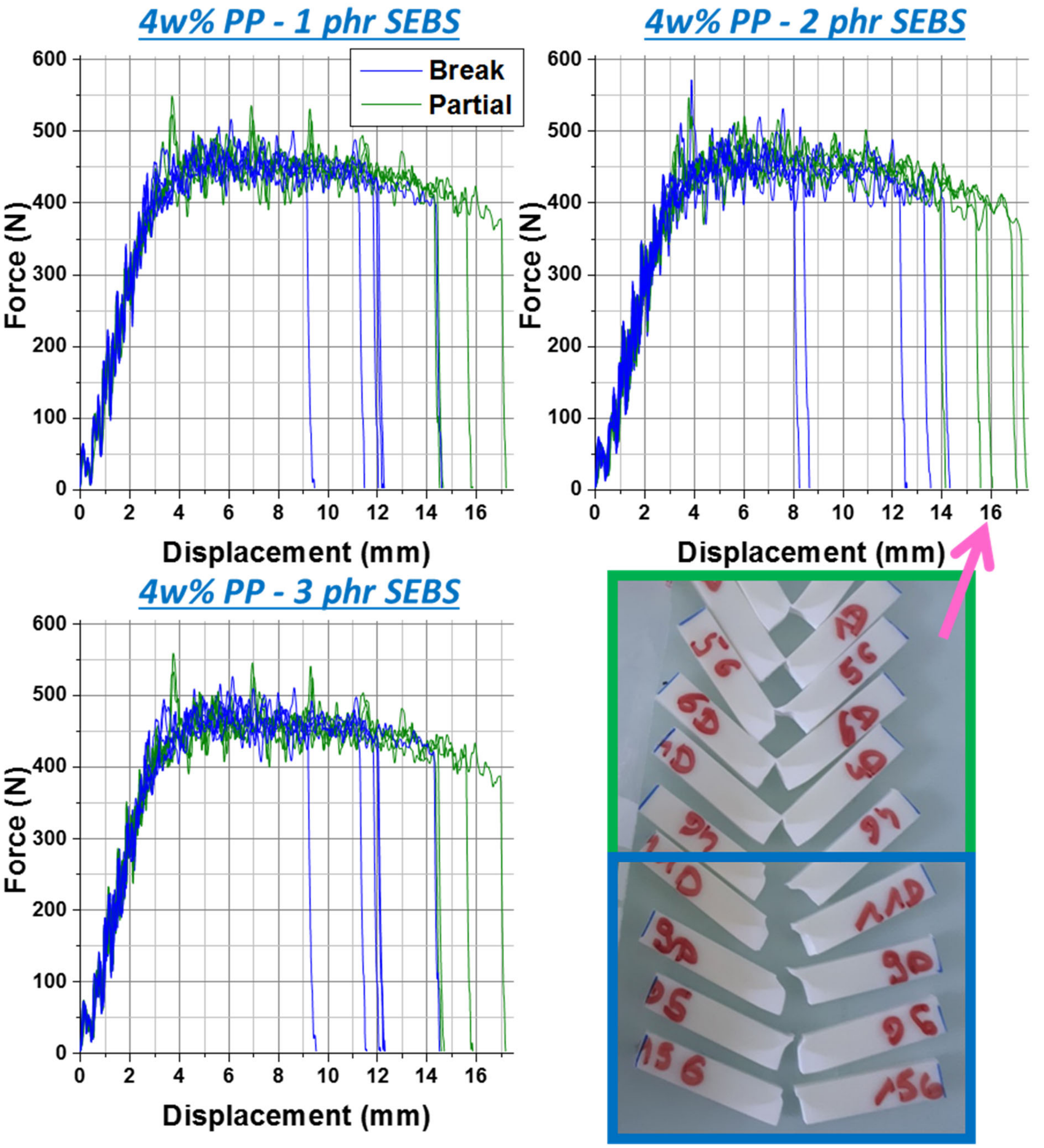

Figure S8: Force-displacement curves of unnotched Charpy impact of ABS contaminated with 4w% PP and additived with 1, 2 & 3 phr SEBS (a plot for each rate) and picture of 2 phr SEBS specimens after impact – green curves and frame for partially broken specimens

3.2.2 Morphology

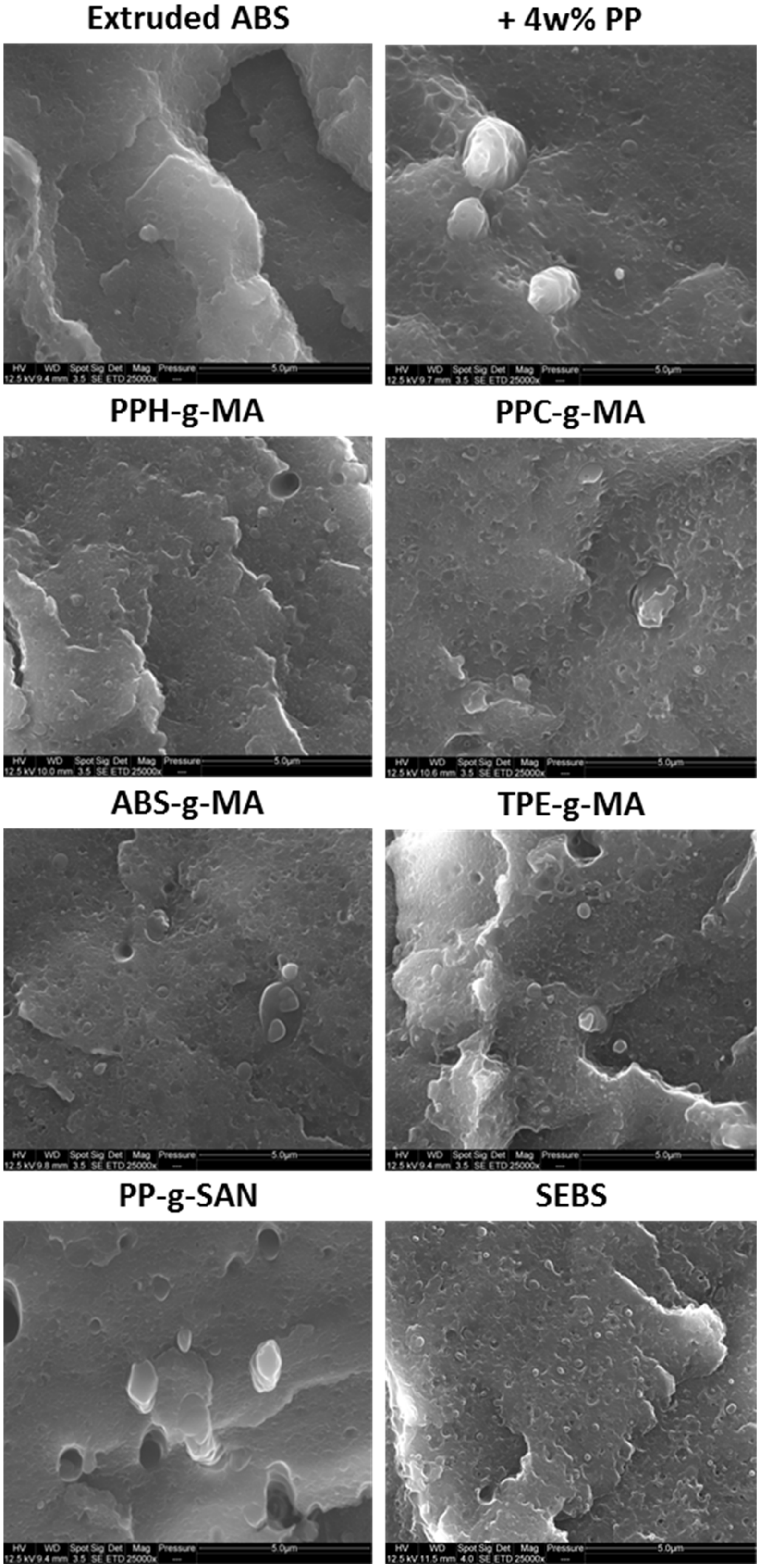

Figure S9: SEM pictures of cryofractured dogbones – effect of compatibilizers at 3 phr on a 4w% PP contamination – 25000 x magnification

3.2.3 Tensile properties

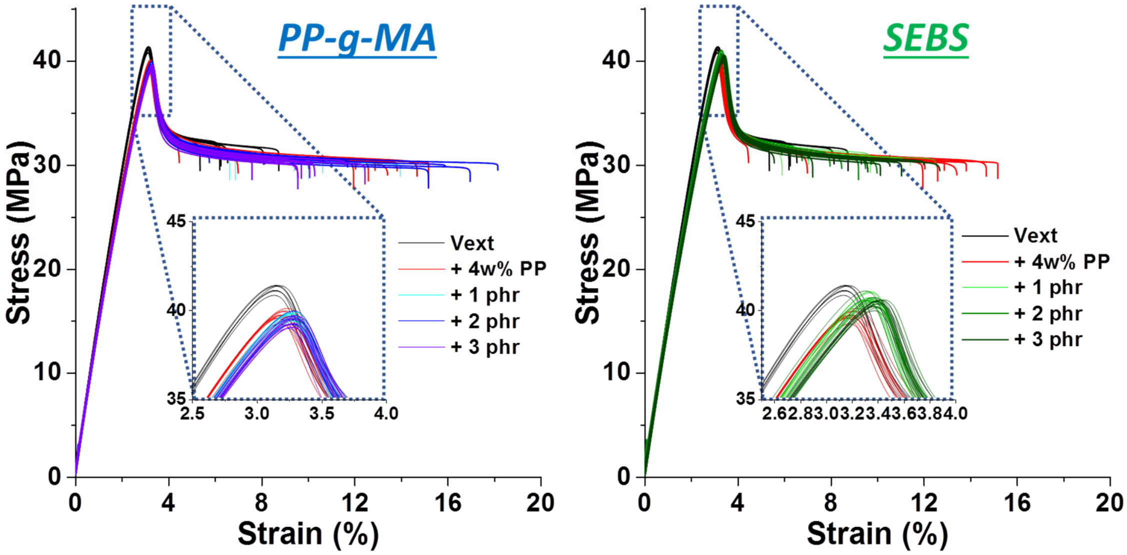

Figure S10: Tensile tests of compatibilization trials with PP-g-MA and SEBS of ABS contaminated with 4w% of PP – magnification on stress peak -"Vext" for virgin and extruded ABS, other batches with 4w% PP – phr rates correspond to PP-g-MA and SEBS

D. Appendices to "3.3 D.O.E.-R.S.M.: Influence of process parameters and interactions with compatibilizers toward impact properties"

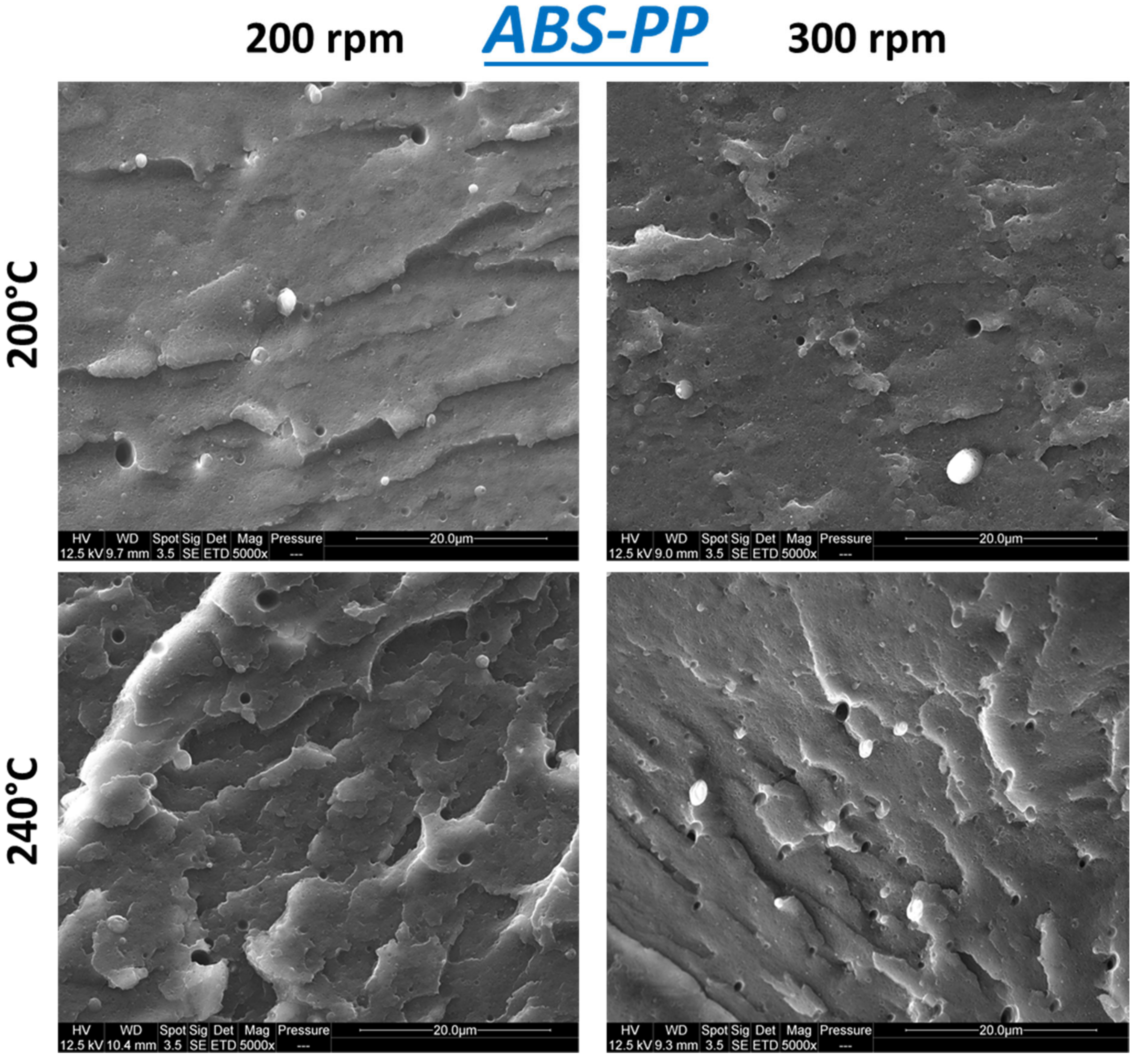

Figure S11: SEM pictures of cryofractured dogbones of ABS contaminated with 4 w% PP – different extrusion temperatures and screw speeds

PP-g-SAN – 200°C – 200 rpm – 0.5 phr

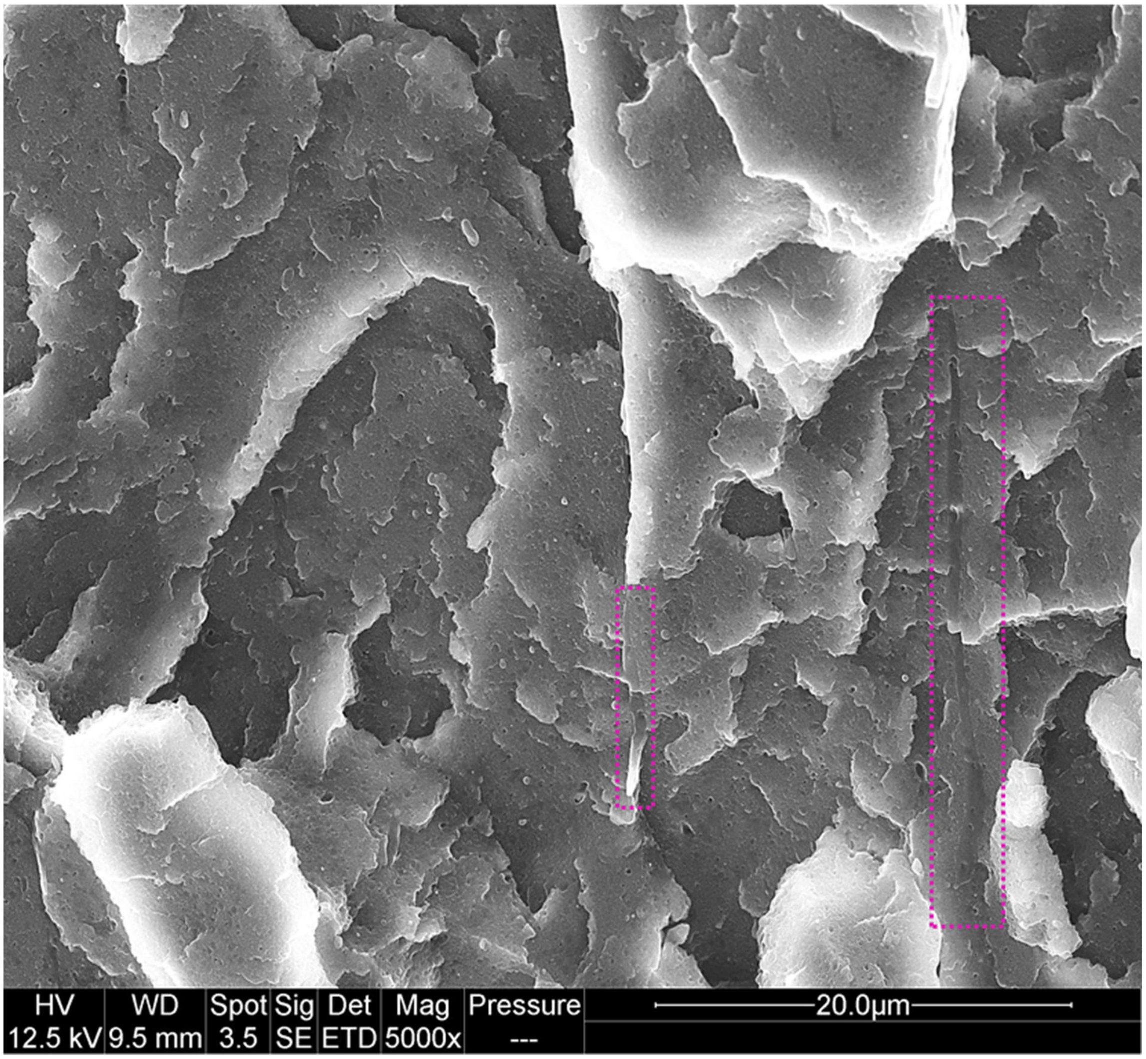

Figure S12: SEM pictures of cryofractured dogbones of ABS + 4 w% PP – 0.5 phr PP-g-SAN, 200°C and 200 rpm – near the surface, elongated phases framed in pink

PP-g-SAN – 240°C – 300 rpm – 0.5 phr

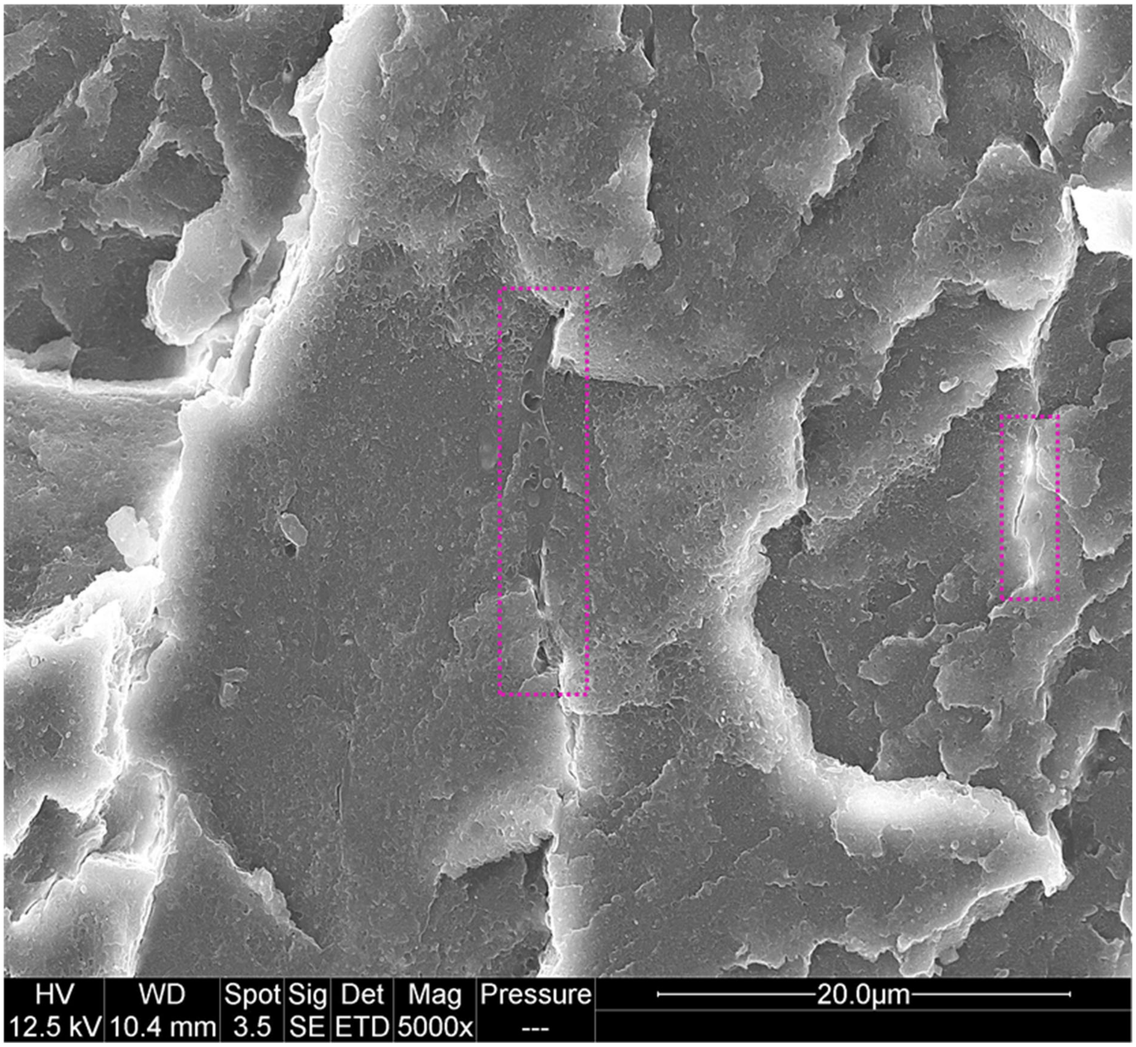

Figure S13: SEM pictures of cryofractured dogbones of ABS + 4 w% PP – 0.5 phr PP-g-SAN, 240°C and 300 rpm – near the surface, elongated phases framed in pink

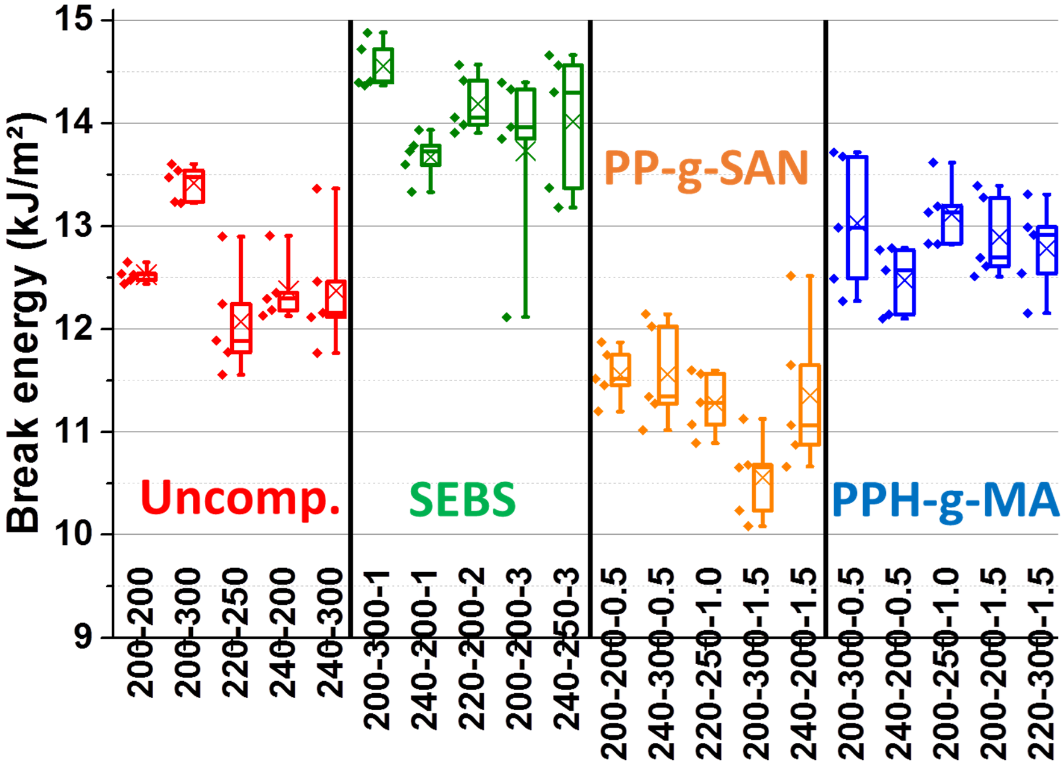

Figure S14: Notched Charpy impact break energies – D.O.E.-R.S.M. experimental values – ABS + 4w% PP without compatibilizer or with SEBS, PP-g-SAN or PP-g-MA – abscissa axis indicates extrusion temperature (°C), screw speed (rpm) and compatibilizer loading rate (phr)

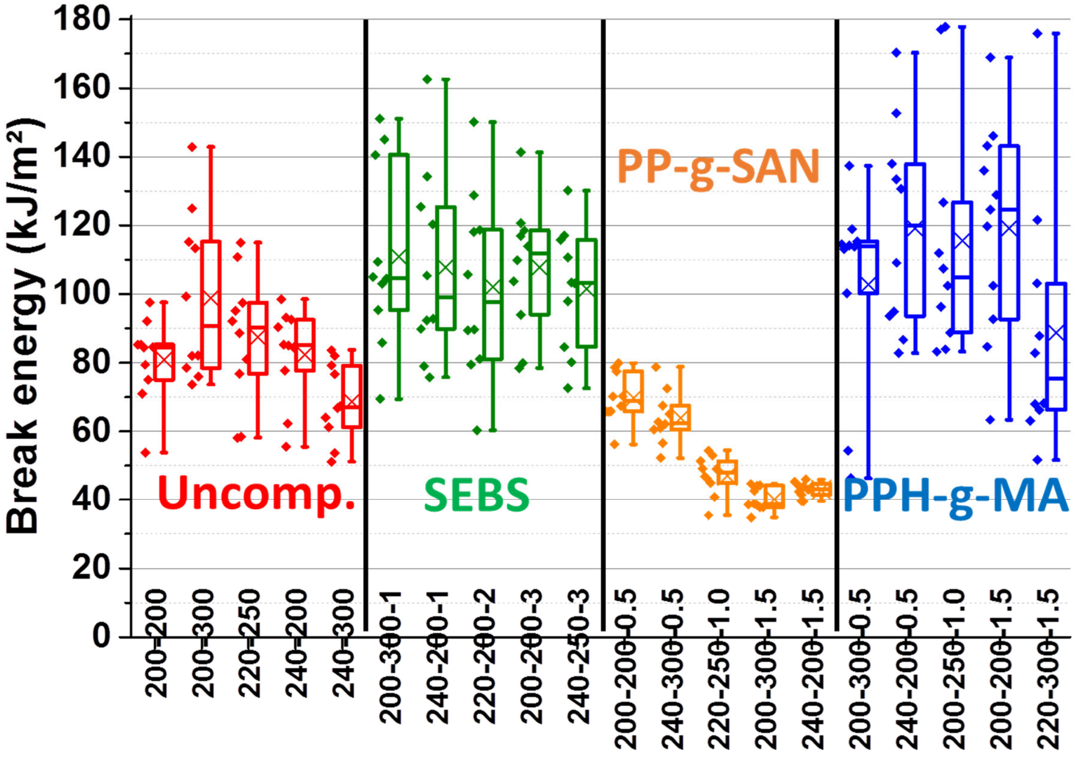

Figure S15: Unnotched Charpy impact break energies – D.O.E.-R.S.M. experimental values – samples and designation identical to previous figure

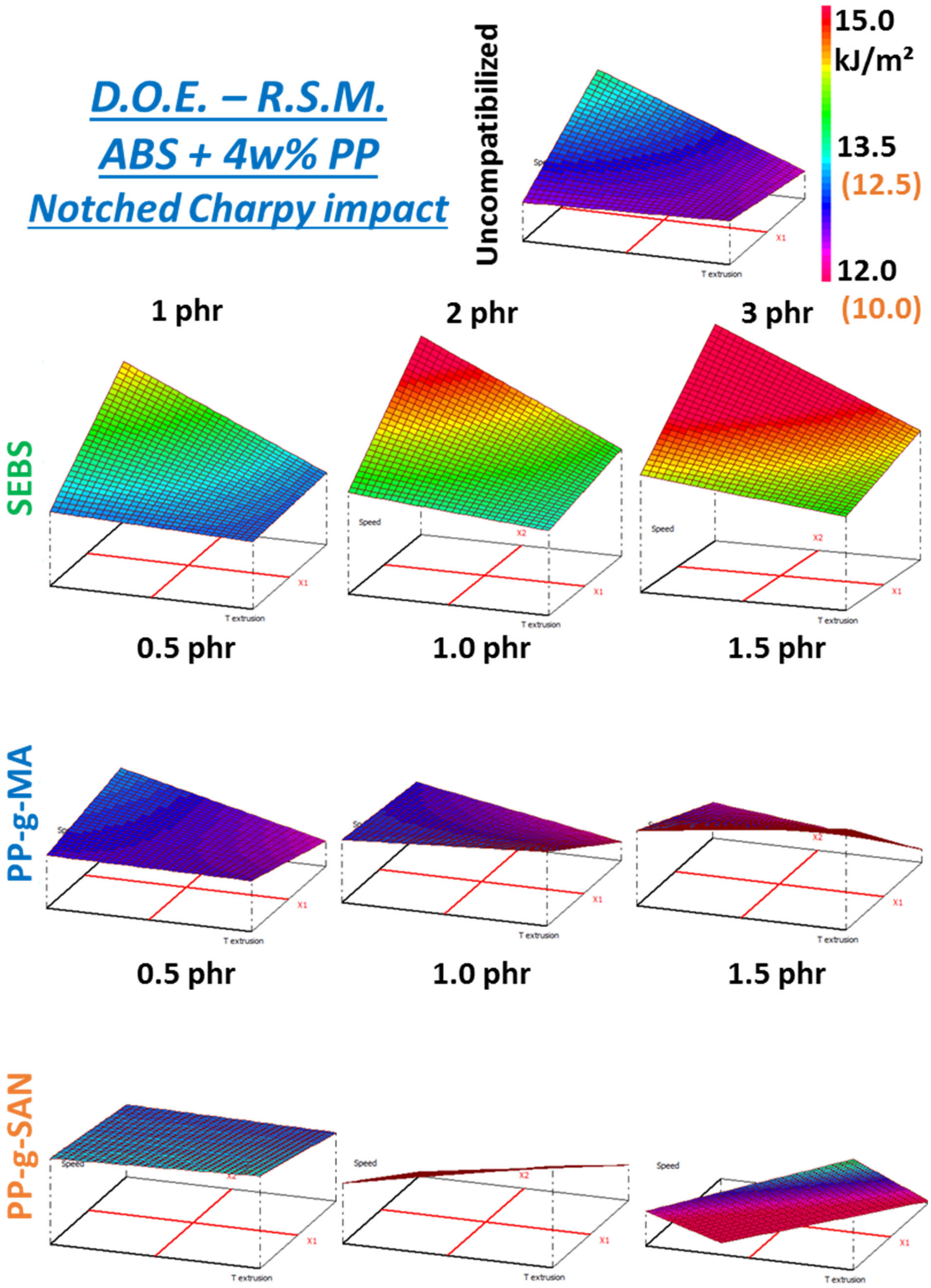

Figure S16: Response Surface Methodology applied to notched Charpy break energy of ABS + 4w% PP system - uncompatibilized, additived with SEBS, PP-g-MA or PP-g-SAN – additive concentration (1-3 or 0.5-1.5 phr), extruder temperature (200-240°C) and screw speed (200-300 rpm) as variables – lower color scale for PP-g-SAN (values in orange)

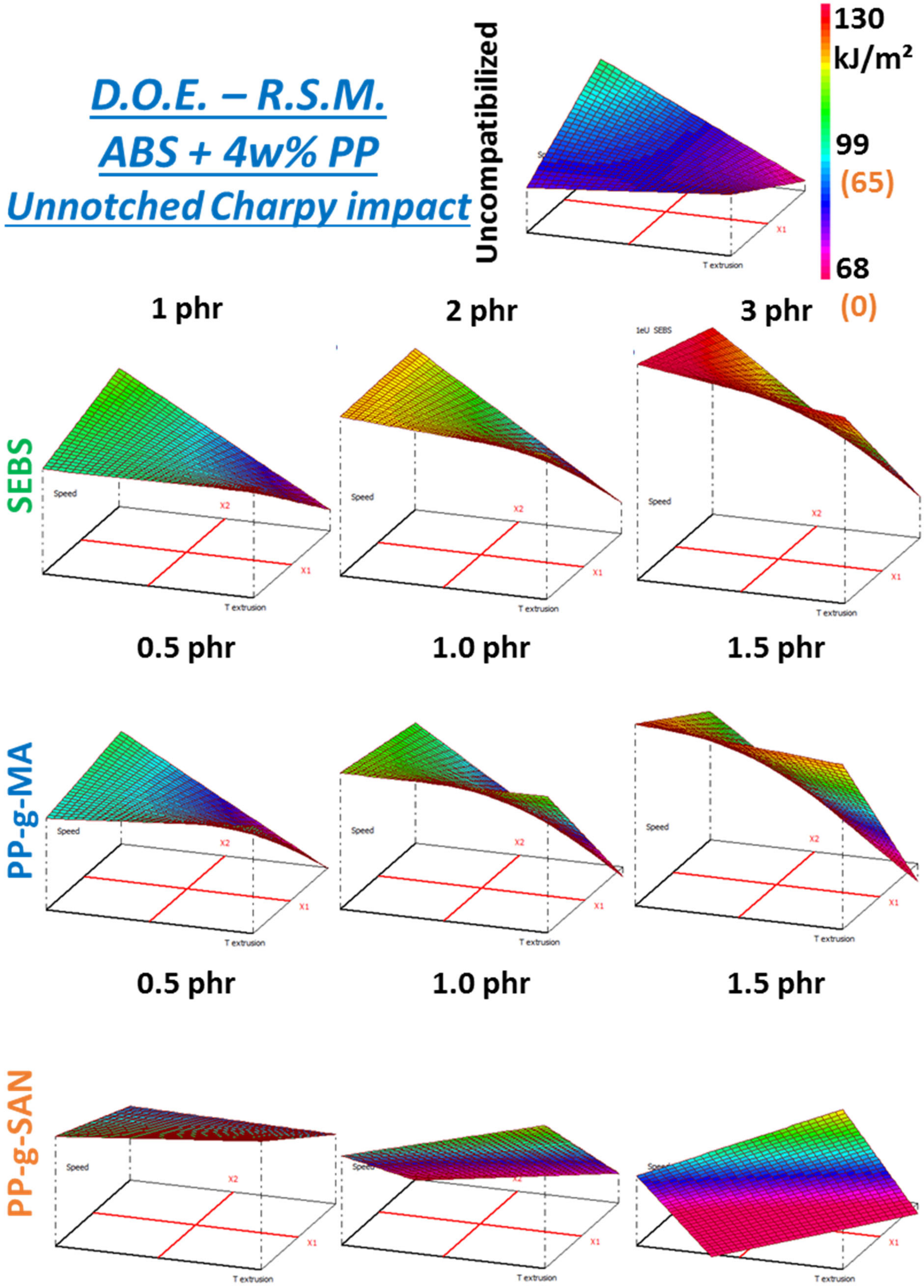

Figure S17: Response Surface Methodology applied to unnotched Charpy break energy of ABS + 4w% PP system - uncompatibilized, additived with SEBS, PP-g-MA or PP-g-SAN – additive concentration (1-3 or 0.5-1.5 phr), extruder temperature (200-240°C) and screw speed (200-300 rpm) as variables – lower color scale for PP-g-SAN (values in orange)
